# Recycling Functional Fillers from Waste Tires for Tailored Polystyrene Composites: Mechanical, Fire Retarding, Electromagnetic Field Shielding, and Acoustic Insulation Properties—A Short Review

**DOI:** 10.3390/ma17112675

**Published:** 2024-06-01

**Authors:** Jinlong Zhang, Hang Liu, Shyam S. Sablani, Qinglin Wu

**Affiliations:** 1 School for Engineering of Matter, Transport and Energy, Arizona State University, Tempe, AZ 85287, USA; 2 Department of Apparel, Merchandising, Design and Textiles, Washington State University, Pullman, WA 99164, USA; hangliu@wsu.edu; 3 Department of Biological System Engineering, Washington State University, Pullman, WA 99164, USA; ssablani@wsu.edu; 4 School of Renewable Natural Resources, Louisiana State University Agricultural Center, Baton Rouge, LA 70803, USA

**Keywords:** waste tire, carbon black, textile fiber, polystyrene, waste rubber recycling

## Abstract

Polymer waste is currently a big and challenging issue throughout the world. Waste tires represent an important source of polymer waste. Therefore, it is highly desirable to recycle functional fillers from waste tires to develop composite materials for advanced applications. The primary theme of this review involves an overview of developing polystyrene (PS) composites using materials from recycled tires as fillers; waste tire recycling in terms of ground tire rubbers, carbon black, and textile fibers; surface treatments of the fillers to optimize various composite properties; and the mechanical, fire retarding, acoustic, and electromagnetic field (EMI) shielding performances of PS composite materials. The development of composite materials from polystyrene and recycled waste tires provides a novel avenue to achieve reductions in carbon emission goals and closed-loop plastic recycling, which is of significance in the development of circular economics and an environmentally friendly society.

## 1. Introduction

Polymers, commonly named plastics, have become widely used as a consequence of their low cost and favorable properties, and it is estimated that by the year 2050 their total production will reach above 500 million tons. This ever-increasing production, combined with our desire for disposability and poor infrastructure, has led to a plague of plastics in our environment [1]. Therefore, plastic pollution is currently a big and challenging issue throughout the world [2,3]. Among the accumulated polymer waste, polystyrene (PS) waste represents around 8% of the total [4]. The incineration of PS plastic waste releases significant carbon dioxide emissions and causes significant environmental pollution. Therefore, designing high-performance PS composites to extend their lifetime has attracted significant interest, aiming to reduce PS waste production and subsequently achieve carbon emission reductions and carbon neutrality [5].

One drawback of PS is its low impact strength, and the toughening of PS for high-performance PS composites is usually performed by blending PS with rubber elastomers, such as ethylene–propylene rubber (EPR), natural rubber, and styrene–butadiene rubber [6]. However, the expense of synthetic rubbers, especially EPR, limits the practical application of these composites, although the mechanical properties of PS can be improved with these rubbers. Therefore, it is of importance to develop new methods to tailor PS properties. Waste tires represent an important source of polymer waste. Ground tire rubber is a recycled product that can be combined with PS to produce high-performance, waste rubber-toughened PS composites [5]. These PS and waste tire rubber composites have potential applications in thermal and acoustic insulation construction materials [7], asphalt binders [8,9], packaging containers [10], and computer display shells. However, waste tire rubber is not compatible with PS due to its three-dimensional cross-linking structure. Surface modifications are needed to improve the interface interactions between rubber particles and the PS matrix [11]. In addition to the rubbers in waste tires, carbon black accounts for about 20 wt% of rubber tires and is an ideal conductive filler to tailor polystyrene composites’ conductivity and anti-static properties. Additionally, composite materials of rubber and polystyrene have low fire retarding properties, so additives are necessary to tailor the fire retardancy of these polystyrene composites [12,13]. Moreover, by-product textile fibers (5–15 wt% of tires [14]) recycled from waste tires make it possible to tailor the acoustic insulation properties of recycled PS composite materials, which has intriguing applications. According to a literature search, some review papers have been published in terms of recycling waste tires via blending them with rubber elastomers [15], thermoplastics [16], thermosets [17], and concrete [18]. However, for review papers on PS and recycled waste tire composite materials, only one publication has been reported, according to our knowledge, but this paper primarily focuses on the compounding parameters of PS/tire rubber powder composites and their mechanical properties [19]. In addition, this review paper has no mentions in terms of recycled carbon black and textile fibers from waste tires or their effect the properties of PS composites, e.g., acoustic, fire retarding, and EMI shielding properties. Although recycled waste tire rubber-modified thermoplastic and thermoset composites, and high-performance PS composites using commercialized fillers instead of recycled tire rubber fillers, have been reviewed, the scope of this review primarily focuses on the development of high-performance PS functional composites via the utilization of different types of recycled functional fillers from waste tires (carbon black, tire rubbers, and textile fibers), especially carbon black and textiles, which are less mentioned in the literature currently. These composite materials made from PS and tire rubbers, carbon black, or textile fibers have potential for use as antistatic packaging materials and engineering wall panels with fire retarding, thermal insulating, and sound-absorbing properties. Therefore, the theme of this short review focuses on the recycling of functional fillers from waste tires for the development of high-performance PS composites with enhanced mechanical, fire retarding, electromagnetic field (EMI) shielding, and acoustic insulation properties.

## 2. Overview of the Recycling of Rubber Powders, Carbon Black, and Textile Fibers from Waste Tires

**Figure 1:**
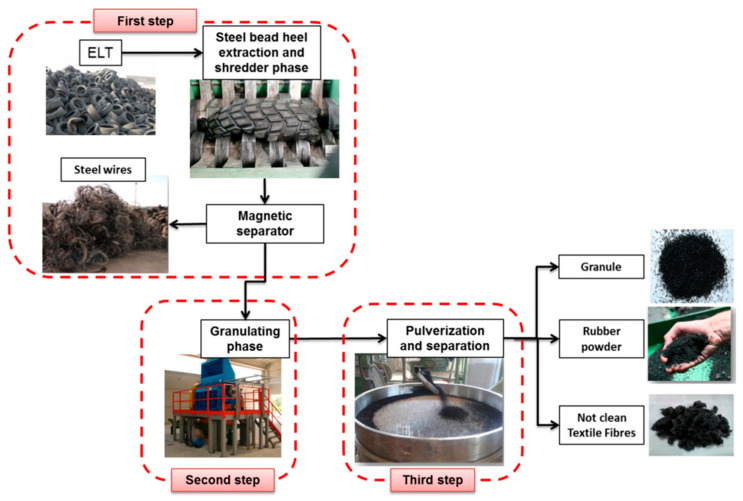
Waste tire recycling process (Landi, et al. [23]). (Figure republished from Zhou et al. [24] (2010) with permission from Elsevier Publishing Co. (Amsterdam, The Netherlands)).

A challenging issue faced by modern society is the constant increase of waste. In particular, approximately 290 million tires per year, as rubber waste, are discarded in the United States. However, these rubber waste products are non-biodegradable. Approximately 2 billion waste tires have accumulated in the United States, and these waste tires in their long-term storage can easily cause health and environmental issues if they are not managed in proper ways. The chemical toxicity and micro- or nano-particles from waste tires have attracted attention, as these particles released in the marine environment are potentially harmful for both ocean species and humans [20,21,22]. Therefore, it is a big challenge to deal with these large amounts of waste. Conversion of waste tires into energy by incineration is a straightforward approach for recycling waste tires, but the emission of sulfur oxides and polycyclic aromatic hydrocarbons resulting from inefficient combustion causes air pollution. High-temperature pyrolysis is an alternative and practical approach, converting recycled waste tires into carbon black, pyrolytic oil, and gases, but the primary limitation of pyrolysis is the generation of toxic hydrogen sulfide gas as a by-product. Therefore, it is necessary to find a feasible solution to recycle and reuse these waste tires. According to the literature, mixed rubber powders, carbon black, and textile fibers account for 90% of waste tires, so recycling of these waste materials is a primary theme [14]. As a practical and widespread approach for recycling waste tires, the grinding process under cryogenic conditions is an environmentally sustainable method of converting waste tires into carbon black, tire rubbers, and textile fibers, which primarily includes three steps, as shown in Figure 1. The waste tires are initially downsized into particles of 7–10 cm via grinder blades and knives, followed by the separation of metallic fractions, in the first step. Tire rubber granulates of about 2 cm are further processed in the second step. Finally, pulverized rubber fractions are produced from tire residues and further separated from the textile fibers and metal components by using pneumatic separators and electromagnets, respectively [23].

Waste tires are composed of about 40–50 wt% of mixed rubbers in Table 1, including styrene–butadiene rubber, natural rubber, and butadiene rubber, which can be ideal elastomers to tailor the mechanical performance of thermoplastic materials. In terms of price, waste tire rubbers have an advantage over new synthetic rubbers for the design of composite materials, especially those from recycled resources. Carbon black, as the second major component, accounts for 20–25 wt% of waste tires in Table 1. Recycled carbon black from tires is composed of mixed carbon black, traces of steel, and inorganic additives (such as calcium carbonate, silicon oxide, and zinc oxide) [4]. They have different surface morphologies, particle sizes, and varied components compared to commercial carbon black. The recycled carbon black usually has an irregular distribution in particle size, and the chemical and morphological properties are obviously different from those of conventional carbon black. The ash contents of recycled carbon black are relatively higher too. Most importantly, the carbonaceous residues produced in the recycled carbon black causes the deactivation and blockage of the carbon’s active sites. Nonetheless, recycled carbon black has potential uses as reinforcing or conductive fillers in the production of polymer composites and as pigments in the saturation of structural color materials. Waste tire textile fibers, as a by-product of waste tires, account for 5–15 wt% in Table 1 [23]. However, the conventional ways of dealing with waste textile fibers, burying and burning, cause ecological and environmental problems. Therefore, the recycling of textile fibers is a challenging issue currently. Polyester and Nylon 6,6 are the main components in waste tire textile fibers [25].

## 3. Surface Treatments of Waste Tire Rubber Powders, Carbon Black, and Textile Fibers

### 3.1. Surface Activation of Recycled Waste Rubber Powders

Due to the low reactive surface of waste tire rubber powders, the miscibility among waste rubbers and thermoplastic resin matrix is low. Therefore, surface modification of waste tire rubber powders is needed for enhancing the reinforcing performance and tailoring composite properties.

Modifying the surface of waste rubber powders is primarily achieved through devulcanization, chemical oxidation, high-energy electron treatment, coating, and grafting polymerization to form polar groups (hydroxyl or carbonyl groups) for better compatibility with thermoplastics. Devulcanization has been used to break the cross-linking structure of waste rubber powders through mills and screw extruders in the presence of devulcanization agents [26]. Thermomechanical and thermochemical devulcanization, as two main methods, have been used to cleave the cross-linking structure of waste rubber powders to improve their compatibility with thermoplastics [27]. Surface functionalization of waste rubber powders has also been carried out by chemical oxidation, in addition to physical modification approaches. Potassium permanganate [28], periodic acid [29], nitric acid [30], sulfuric acid [31], and hyperchloric acid [32] have been used to oxidize the surface of waste rubber powders for better interfacial adhesion with thermoplastic matrixes. Moreover, the surface of waste rubber powders has been activated by high energy radiation, such as gamma radiation, plasma, and corona radiation, in addition to electron beam radiation [30,33,34]. These methods bring about better interfacial adhesion of waste rubber powders and thermoplastic matrixes compared to neat waste tire powder composites [34]. Furthermore, surface activation of waste rubber powders via coatings of ethylene acrylic copolymer or maleated polypropylene has been introduced, and the precoated waste rubber powders were uniformly dispersed in a thermoplastic matrix [35,36]. Grafting polymerization, as another chemical modification approach, can selectively modify the surface of waste rubber powders. Functionalization of waste rubber powders has been studied through free-radical initiation or photo-initiation grafting polymerization in the presence of initiators and monomers. The most commonly used initiators for free-radical polymerization are benzol and dicumyl peroxide, while ultraviolet energy is usually used for photo-initiation polymerization in the presence of photo initiators or photosensitizers [37]. The aggregates of waste rubber powders are reported to be reduced after grafting polymerization and, therefore, the interfacial adhesion of waste rubber powder and thermoplastics is improved [37]. Therefore, the surface of waste tire rubber powders is activated after physical and chemical modifications, and the enhanced interfacial miscibility of modified waste tire rubbers and thermoplastic matrixes promotes certain mechanical properties in the resulting waste tire rubber composites, e.g., styrene-grafted waste tire rubber/polystyrene resin composites.

### 3.2. Surface Modification of Recycled Carbon Black

**Figure 2:**
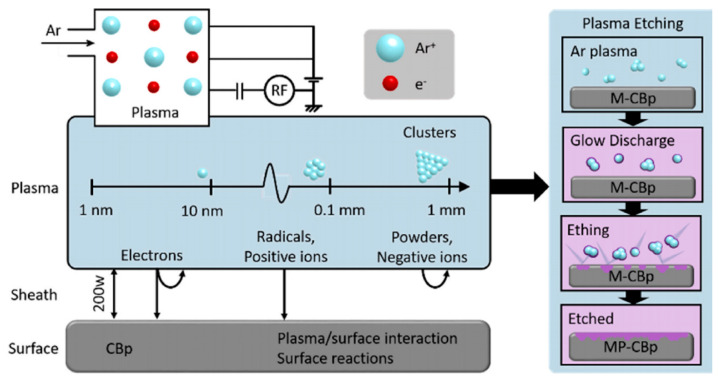
Plasma apparatus for surface activities of carbon black (Zhao et al. [41]). (Figure republished from Valentini et al. [42] (2020) with permission from Elsevier Publishing Co.).

The carbon black derived from waste tires is much different from commercial carbon black. It is composed of large particle sizes, inertia surface activities, and high mineral or inorganic contents. Therefore, it is necessary to tailor its surface properties for carbon black composite materials. With mineral or inorganic residues on the surface of recycled carbon black, demineralization with a bases or acids is a necessary step to activate its surface. Removal of impurities from its pyrolytic char is usually conducted by concentrated acids. For instance, removal of minerals was investigated via the acid treatment method on recycled carbon black surfaces [38]. A demineralization rate of up to over 90% with hydrochloric acid and hydrogen fluoride mixed acids is further reported [24]. Recycled carbon black treated by nitric acid also makes it possible to remove the deposition of carbonaceous and inorganic materials [39]. However, the waste gas and acid produced during these harsh acid treatments leads to environmental pollution. To further optimize the acid treatment protocols, sulfuric acid and sodium hydroxide treatments of recycled carbon black have been investigated [40], and its surface areas are enlarged, in addition to the improvement of its structures. Reusing alkali and acid solutions also reduces the waste gas emissions to some degree, while the environmental pollution issues cannot be completely addressed. Thus, it is highly desirable to develop an efficient and clean technology to remove impurities for utilizing recycled carbon black at high efficiency, e.g., plasma. For instance, plasma treatment with three carrier gases has been investigated to improve the surface activities of recycled carbon black and reduce its average particle size, as shown in Figure 2 [41].

### 3.3. Surface Treatments of Recycled Textile Fibers

As the recycling of textile fibers from waste tires is in its infancy, few works about surface treatments of textile fibers for tailoring the interfacial miscibility of their composites have been published. Like surface treatments of waste tire rubbers, acid oxidation treatments have been studied to tailor textile fiber surface properties. Physical plasma treatment technologies are also utilized to enhance surface properties, in addition to chemical modifications. For instance, free radicals produced via plasma treatments of textile fibers lead to the production of functional groups and form bonds between fibers and thermoplastic matrixes [43].

## 4. Waste Tire-Derived, Recycled Filler-Tailored PS Composites

### 4.1. Mechanical Properties

PS is brittle, with relatively low impact strength compared to other thermoplastics, such as high-impact polystyrene and acrylonitrile butadiene styrene. Different types of synthetic rubbers, such as styrene–butadiene rubber (SBR), natural rubber (NR), ethylene–propylene–diene monomer (EPDM), and ethylene–propylene rubber (EPR), have been studied to improve the mechanical properties of PS. EPDM elastomers have been used to toughen PS via in situ polymerizations and blends. The impact strength of a PS-grafted EPDM copolymer synthesized via a bulk polymerization technique was increased by 80–170% compared to that of PS [44]. The increased impact strength of PS/EPDM blends was also achieved after the addition of divinylbenzene, trimethylolpropane triacrylate, and styrene–butadiene–styrene block copolymers [45,46]. For toughening PS with NR, the main concerns are about compatibilizers and processing techniques. The compatibility of PS/NR blends was improved after the addition of a compatibilizer PS-grafted NR copolymer [47]. In addition, using SBR and EPR elastomers to toughen PS has been studied as well. The impact strength of PS was improved with the addition of a SBR-grafted PS copolymer [48]. The toughness of PS/EPR blends was also improved after the addition of compatibilizers [49]. However, the expense of synthetic rubbers, especially EPDM and EPR, limits the practical application of these composites, although the mechanical properties of PS have been improved with these rubber elastomers. Therefore, it is of importance to develop new methods to improve the mechanical properties of PS.

The waste tire-derived rubber powders are ideal elastomers for tailoring the mechanical properties of PS materials, especially impact strength. The mechanical properties of waste tire rubbers and PS composites in the literature are summarized in Table 2. Two main strategies have been studied to tailor the mechanical properties and miscibility of PS and waste tire rubber powder composites; namely, the addition of compatibilizers and the surface modification of waste tire rubber powders. For the compatibilizer method, compatibilizers are physically blended with waste rubber powders and thermoplastics to improve the compatibility of components. For instance, polystyrene grafted styrene–butadiene rubber was synthesized via emulsion polymerization and then used as the compatibilizer in waste tire rubber powder and PS blends [6]. The interfacial adhesion of blends was improved with a typical discontinuous–continuous morphology, and enhanced tensile strength and impact strength were achieved via the addition of compatibilizers. Similarly, styrene–butadiene–styrene block copolymer, as a compatibilizer, was used for high-impact polystyrene/ethylene vinyl acetate copolymer/waste rubber powder composites [50]. Another way is by the surface modification of waste tire rubber powders. For example, waste tire rubber powders were graft-modified with styrene by the conventional radical polymerization method [51]. The impact strength of waste tire rubber and PS blends with a content of styrene–g–waste tire rubber of 25 wt% had a nearly 4-fold increase compared to that of neat PS. It can be explained that the polar groups of graft-modified rubber powder by styrene were similar to that of PS, and the main role of grafted short-chain PS was to act as an interfacial agent which contributed to the improved compatibility between waste tire rubber powder and PS.

### 4.2. Fire Retarding Properties

Low fire retarding performance is another issue of PS, as it is not a fire resistant material with a low limited oxygen index (below 21%). The fire retardant performance of PS can be improved by the addition of flame retardants. The most effective flame retardants for PS are halogen-containing flame retardants [58]. For instance, poly(decabrominated diphenyl ethers) and tetrabromobisphenol-A have been used [59,60]. However, they usually release toxic gases and heavy smoke during combustion, so halogen-containing flame retardant is not preferred at present due to environmental concerns. Therefore, development of environmentally friendly halogen-free flame retardant is a new direction for PS. Additive-type and reactive-type environmentally friendly halogen-free flame retardants are examples. Although reactive-type flame retardants have better thermal stability and low toxicity, their complex processing techniques and expense limit their wide utilization. Therefore, additive-type flame retardants (inorganic and organic additive-type flame retardant), with relatively low prices and simple processing techniques, are popular for improving the flame retarding of PS.

Inorganic additive-type flame retardants have unique merits, in terms of better thermal stability, low toxicity, and less emission of corrosive gases, in addition to their low price. Montmorillonite, metal hydroxides (magnesium hydroxide and aluminum hydroxide), and carbon black have been widely studied for PS fire resistance [61]. Compared to commercialized carbon black, recycled carbon black derived from waste tires is ideal for tailoring the fire retarding performance of PS materials. For instance, the limited oxygen indexes of natural rubber (NR) and polypropylene (PP) increased (LOI) from 26 to 32 for NR and 17.5 to 25.5 for PP after the addition of 5 wt% carbon black. The increased fire retarding performance of an ethylene–vinyl acetate (EVA) composite with waste carbon black derived from waste tires was also achieved. The possible fire retarding mechanism is attributed to the scavenging of peroxy radicals by carbon black and a barrier established by the oxidation cross-linking of the polymer matrix against both mass and heat transfer [62]. Although better fire performance of PS can be achieved, a high loading of an inorganic additive-type flame retardant is required, which deteriorates the mechanical properties of composites. Thus, the development of new fire additives is a priority for achieving a trade-off in the mechanical and fire retarding performance of PS materials. A synergistic flame retarding strategy enables better flame retarding for PS. Recently, polydopamine was reported as a fire-retardant additive for polymer composites [63]. Therefore, polydopamine-coated carbon black and phytic acid-tailored carbon black are promising additives for PS composites in terms of smoke releasing reduction and fire retarding improvement [64].

### 4.3. Conductivity and EMI Shielding Properties

**Figure 3:**
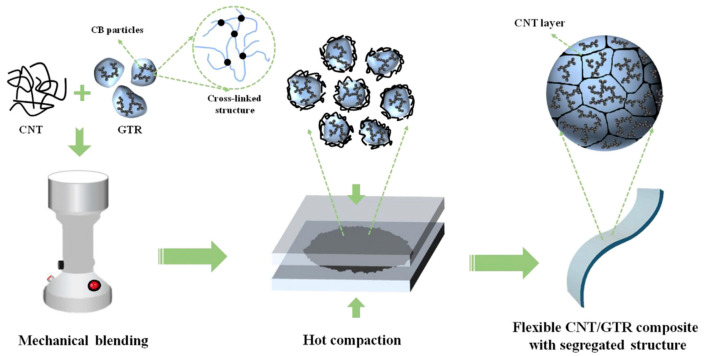
Fabrication of flexible carbon nanotube/ground tire rubber composites with segregated structure via mechanical blending and hot-press melting compounding (Jia et al. [68]). (Figure republished from Zhang et al. [63] (2020) with permission from Elsevier Publishing Co.).

With the advent of the 5G era, electromagnetic radiation pollution has received increasing attention. Therefore, it is highly demanded to develop PS materials with excellent EMI shielding performance to expand their applications. An excellent EMI shielding efficiency is highly dependent on conductive fillers and conductive network structures. The primary carbon fillers used to tailor the EMI shielding performance of PS materials are metals (Cu, Al, etc.) and carbon fillers, such as carbon nanotubes, carbon fibers, and carbon black. Compared to the EMI shielding performance of carbon fibers and carbon nanotubes, carbon black has unique advantages in terms of its low price and widely available sources. Therefore, carbon black as a conductive filler is frequently studied in conductive PS composite materials [65]. Carbon black accounts for about 20% in waste tires and is an ideal alternative for commercial carbon black for tailoring the conductivity properties of PS composites. However, a high content of carbon black is required to achieve excellent conductivity and EMI shielding performance. The high loading of conductive carbon black causes difficulty its dispersion and aggregation in the resulting composite materials, in addition to obviously decreased mechanical properties [66]. Additionally, a high loading of conductive fillers raises composite processing issues. Therefore, it is urgent to investigate and develop PS composite materials with high conductivity and outstanding EMI shielding performance with the prerequisite of relatively low loadings of carbon black. The EMI shielding performance of carbon black and PS composites is highly dependent on establishing a conductive network structure, but it is a challenging issue to establish conductive network structures to achieve a satisfying EMI shielding performance of carbon black-tailored PS composite materials [67]. The primary approaches to designing the continuously conductive network structures in carbon black composites involve emulsion templates, aerogel templates, and 3D printing. For the emulsion template method, PS emulsion particles work as the template and carbon black conductive fillers function as the conductivity media. The conductive fillers are encapsulated on the surface of PS particles via the self-assembly of carbon black conductive fillers and PS emulsion particles. The organized micro- or nano-structure conductive network is then established in the carbon black and PS composite material. Most importantly, the emulsion template strategy enables the effective establishment of the conductive network structures with a relatively low loading of carbon black conductive fillers. Another promising approach to achieving a continuously conductive network structure established with a low level of conductive fillers is the aerogel template method. Namely, a conductive carbon black aerogel material works as the template, and a three-dimensional conductive network structure is then established via the casting approach. Due to the uniquely continuous and porous network structures of the conductive frameworks in carbon black aerogels, the ions and electrons can easily transport inside and impart composites with excellent conductivity efficiency. In addition, 3D printing is another way to achieve a unique three-dimensional conductive network structural design. Due to the technique limitations, in terms of the recycling of carbon black from waste tires, studies about the conductivity and EMI shielding properties of recycled carbon black composites are still less reported currently. Therefore, of almost all previous studies on the mechanical properties of recycled carbon black composite materials, few reports deal with their EMI shielding and electrical conductivity performance. The electrical conductivity of waste tire rubber powders containing carbon black is about 4.8 × 10^−7^ S/m [68]. Most importantly, the addition of waste tires achieves the goal of the reduction of loading of conductive fillers in conductive composites, as the carbon black in the recycled tire powders constructs a unique conductive channel, thereby improving the overall performance of the resultant carbon black composites, as shown in Figure 3. Therefore, the addition of waste tire rubber powders makes it possible to tailor the electromagnetic shielding and electrically conductive performance of composite materials. For instance, the electrical conductivity of PS composites increased from 6.7 × 10^−14^ to 2 × 10^−14^ S/m as waste tire loading was at 5 wt%, while PS composites with waste tire loading at 70 wt% had an obvious increase in electric conductivity, reaching 4 × 10^−13^ S/m. [55]. In addition to carbon black, the trace amounts of metals in waste tire-derived textile fibers also contribute to the conductivity and EMI shielding performance. The coated trace metals in waste tire rubber and textile fibers are a potential functional filler for tailoring the conductivity and EMI shielding performance of PS composites. However, PS/metal composites derived from waste tires have almost no reports, as the textile fibers recycled from waste tires only began to receive attention a few years ago.

### 4.4. Acoustic Properties

Noise pollution causes serious health issues, resulting from rapid urbanization and industrialization, such as high blood pressure and stress. Therefore, it is urgent to improve the sound insulation properties of PS composite materials. The acoustic characterization of materials is based on their sound absorption coefficient. It is defined as the absorbed energy in the absorbent divided by incident energy [69]. The sound absorption can be calculated by the tube method according to the International Standard ISO10534 [70]. Natural fiber-reinforced polymer composites are of high interest for achieving excellent acoustic properties at an affordable cost. For instance, variations in the noise absorption coefficient of 0.2 were reached as the fine polyester fiber content in polyester composites increased from 0 to 60 wt% [71]. Compared to the acoustic absorption and insulation of polymer composites tailored by natural and synthetic fibers, the recycled textile fibers from waste tires are attracting attention as an alternative for the development of acoustic and insulation composite materials [72].

**Figure 4:**
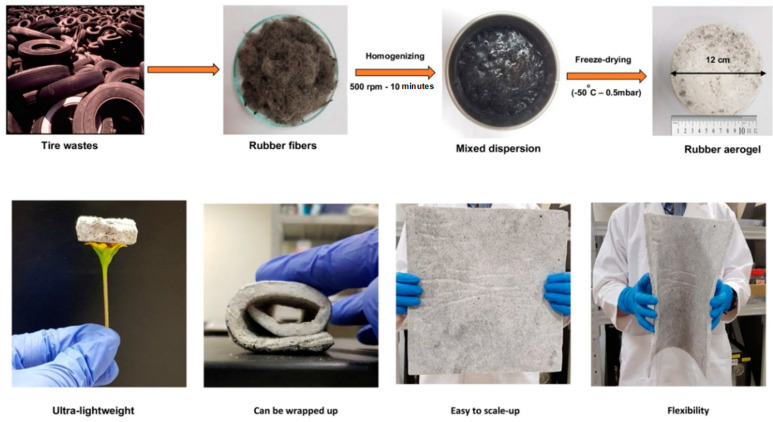
Mass-scale fabrication of sound insulation and lightweight aerogels from waste tire textile fibers via a freeze−drying method (Thai et al. [77]). (Figure republished from Grammelis et al. [14] (2021) with permission from Elsevier Publishing Co.).

The use of waste tire textile fibers as absorbers has been studied in composite panels, and the resulting composite panel materials with tire textile fibers had reduced acoustic coefficients [73,74]. The trace amounts of metals in waste tire-derived textile fibers influence the acoustic performance of their composite materials in sustainable building applications. The tiny amounts of impurities also decrease their sound absorption coefficient in insulation building applications [75]. PS and polyurethane (PU) foams are primarily for thermal insulation building components. However, their sound insulation properties need to be improved. For instance, the noise reduction coefficient of waste textile fiber-tailored PU composite foam materials was increased twice compared to that of neat PU foam materials [76]. In addition to foam materials, aerogels produced via freeze-drying and supercritical carbon dioxide have unique properties. For instance, waste tire textile fiber-derived aerogel materials cross-linked by poly(vinyl alcohol) had excellent sound insulation properties, as shown in Figure 4 [77,78].

## 5. Conclusions and Future Prospects

This review summarized the recycling of the main components of waste tires: rubber mixture powders, carbon black, and textile fibers. Different surface treatment methods used to tailor their surface activation for enhanced miscibility between PS and recycled functional fillers are also summarized. Additionally, the functionality of PS composite materials, in terms of their fire retarding, mechanical, EMI shielding, and acoustic properties, was systemically studied as well. The recycling of tire waste textile fibers is still in its infancy, with few works being reported. However, recycled tire textile fibers are ideal functional fillers to tailor PS composite materials’ mechanical, tribological, and acoustic properties. Recycled carbon black is a conductive filler, but the current literature work is still focused on its reinforcement in composite materials, and the conductivity composite research needs further development, especially in the structural, color, energy, and battery fields, e.g., carbon/quantum dots [79,80,81,82,83]. Waste tire-derived carbon black is an ideal anode material for lithium- or potassium-ion batteries [79,84,85] and makes it possible, as an alternative to commercialized carbon materials, for them to achieve comparative electrochemical performance, e.g., long-term stability and reversible capacity. In addition, carbon dots converted from waste tires are desirable for applications in room temperature phosphorescence materials and fluorescent sensors [82,86,87]. Further, recycled carbon black combined with cellulose microfibril or nanocellulose are ideal materials for oil well cement and for drilling muds or fluids in the oil and gas industry [88,89,90]. Our pioneering work was conducted in terms of high-temperature or pressure drilling fluids combined with rheo-synchrotron small-angle X-ray scattering (Louisiana State University, Baton Rouge, LA, USA), rheo-small-angle neutron scattering (Oak Ridge National Laboratory, Oak Ridge, TN, USA), and machine learning techniques [78,91]. However, the development of aqueous dispersions of carbon black is currently still a big barrier for its mass-scale application in oil well drilling materials. Inspired by the low-friction properties of carbon black/polydimethylsiloxane composite cable materials, recycled carbon black is also an ideal lubricant material. The development of sustainable tribology in elastomer sealants, cables, or bearing materials for applications in extreme environmental conditions has attracted attention via combined low- or high-temperature tribometers with in situ or ex situ synchrotron small-angle X-ray scattering and aberration-corrected transmission electron microscope equipment (Argonne National Laboratory, Lemont, IL, USA and Brookhaven National Laboratory, Long Island, NY, USA) [4,92,93,94]. These materials primarily serve the US military (fighter aircraft or aircraft carriers), US National Aeronautics and Space Administration, or Space Exploration Technologies Corporation, SpaceX (Hawthorne, CA, USA).

As tires have four basic components, rubbers, textiles, carbon black, and metals, the process of recycling waste tires, shown in Figure 1, is complex. The recycling of waste tires via the grinding process under cryogenic conditions is a practical and sustainable approach, as the utilization of cryogenic techniques makes it possible to reduce the energy consumption of grinding and of further separating the reinforcing elements of textile fibers, carbon black, and metals from tire rubber powders. However, it is required to maintain an efficient low temperature in the machine chamber for the grinding process to recycle waste tires, and the efficient separation of textile fibers from metals also poses a technique barrier [95]. Therefore, some challenges and technique barriers need to be addressed before recycling waste tires can be achieved at the industry level.

## Figures and Tables

**Table 1:** Typical compositions of tires [14].

Composition	Passenger Cars	Trucks
Rubber	47%	45%
Carbon Black	21.5%	22%
Fiber	5.5%	-
Steel	16.5%	25%
Zinc Oxide	1%	2%
Additives	7.5%	5%

**Table 2:** Mechanical properties of PS and recycled tire rubber composites.

Rubber Content of PS Composites	Impact Strength	Tensile Strength	Bending Strength	Hardness	Elongation at Break
EPS-Tire Rubber [52] 10 wt% ^a^ 30 wt% ^a^	+ 35% ^b^ 55% ^b^	+ 40% ^b^ 8% ^b^	NA NA NA	NA NA NA	+ 27% ^b^ 50% ^b^
PS-Waste SBR [53] 20 wt% ^a^ 50 wt% ^a^	+ 38% ^b^ 65% ^b^	− 26% ^b^ 59% ^b^	NA NA NA	− 5% ^b^ 12% ^b^	NA NA NA
HIPS-Tire Rubber [54] 10 wt% ^a^ 30 wt% ^a^	− 66% ^b^ 76% ^b^	− 44% ^b^ 50% ^b^	− 59% ^b^ 46% ^b^	NA NA NA	− 90% ^b^ 86% ^b^
PS-Tire Rubber [55] 5 wt% ^a^ 10 wt% ^a^	− 61% ^b^ 69% ^b^	− 18% ^b^ 26% ^b^	NA NA NA	NA NA NA	− 34% ^b^ 43% ^b^
PS-Tire Rubber [42] 20 wt% ^a^ 60 wt% ^a^	+ 76% ^b^ 75% ^b^	− 36% ^b^ 72% ^b^	NA NA NA	NA NA NA	+ 13% ^b^ 23% ^b^
HIPS-Waste SBR [56] 40 wt% ^a^ 80 wt% ^a^	NA NA NA	− 46% ^b^ 63% ^b^	NA NA NA	Constant Constant Constant	+ 72% ^b^ 92% ^b^
ABS-Waste Tire [57] 20 wt% ^a^ 50 wt% ^a^ PS-Tire Rubber [6] 10 wt% ^a^ 30 wt% ^a^	NA NA NA + 33% ^b^ 20% ^b^	− 40% ^b^ 78% ^b^ − 36% ^b^ 58% ^b^	NA NA NA NA NA NA	NA NA NA NA NA NA	− 23% ^b^ 26% ^b^ NA NA NA

EPS: expanded polystyrene; HIPS: high-impact polystyrene; ABS: acrylonitrile–butadiene–styrene; NA: no data; ^a^ tire rubber content in PS composites; ^b^ percentage increase or decrease in mechanical properties of PS/tire rubber composites; +: increase; −: decrease.
